# Single-incision laparoscopic splenectomy in children with massive splenomegaly: A prospective, monocentric pilot study

**DOI:** 10.3389/fped.2022.1097416

**Published:** 2023-01-10

**Authors:** Congjun Wang, Cheng Su, Chao Chen, Xianming Tang, Hong Wang, Wei Li, Yanqiang Li, Qiang Liu, Peng Chen, Yong Li, Yige Luo

**Affiliations:** Department of Pediatric Surgery, The First Affiliated Hospital of Guangxi Medical University, Guangxi Medical University, Nanning, China

**Keywords:** splenectomy, splenomegaly, single-incision laparoscopy, pediatric surgery, surgical outcomes

## Abstract

**Background:**

Single-incision laparoscopic splenectomy (SILS) remains a challenging procedure because of the technical difficulty. In this prospective study, we aimed to evaluate the efficacy and safety of SILS in children with massive splenomegaly.

**Methods:**

Pediatric patients with massive splenomegaly were recruited for SILS in a university-affiliated hospital. The data on patient demographics, clinical features, operative variables, and perioperative outcomes were collected prospectively and analyzed. According to the different surgical instruments, the patients were randomly assigned into two groups: the SILS with straight surgical instrument (SILS-S) group and the SILS with curved surgical instrument (SILS-C) group. A two-group comparative analysis was conducted using perioperative data from the different surgical instrumentation systems.

**Results:**

A total of 120 patients were included, of which 103 patients (success group, 85.83%) had complete SILS, the other 17 (failure group, 14.17%) patients were converted to open (*n* = 4, 3.33%) or multi-incision laparoscopic surgery (*n* = 13, 10.83%). The major cause for surgical failure is uncontrollable bleeding (*n* = 14, 82.35%), and age, height, and weight were the risk factors for failure of SILS, but none of the parameters were independent risk factors. The blood loss in the success group was less than that in the failure group, but no significant differences in other operative and outcome indicators. For SILS, the mean (±SD) operative time was 188 (±48.70) minutes, the median intraoperative blood loss (min, max) was 20 (5, 290) ml, the mean (±SD) time of first anal exhaust was 23.9 (±7.73) hours, and the mean (±SD) postoperative hospital stay was 4.72 (±1.03) days. The median pain score was 3 on 1 day, and 1 on 3 days after the operation. Postoperative complications were identified in 8 (7.77%) cases. However, there were no peri-operative deaths in this series. The SILS-C group had a significantly shorter operation time than the SILS-S group (mean ± SD, 172 ± 44.21 vs. 205 ± 47.90 min). There were no significant differences between the two groups in other perioperative data (*P* < 0.05).

**Conclusion:**

SILS is a safe and feasible treatment in pediatric patients with massive splenomegaly, and curved surgical instrumentation has contributed to developing surgical manipulation.

## Introduction

**Figure 1:**
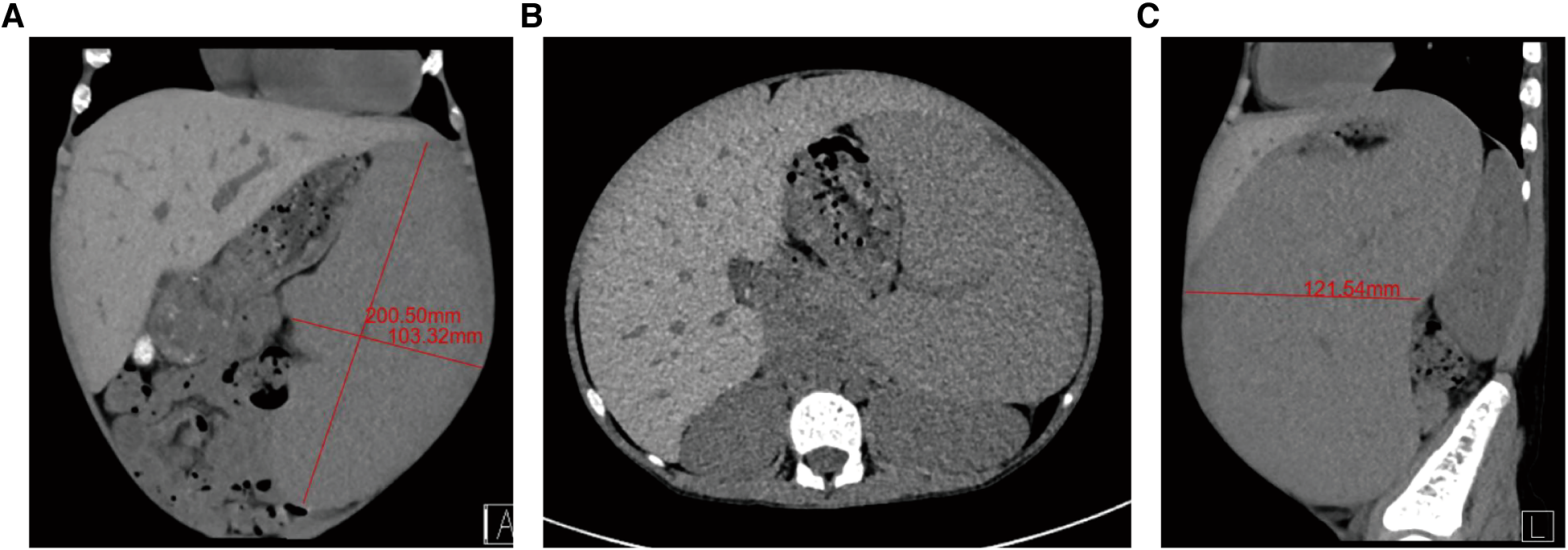
Preoperative computed tomography (CT) images from study participants. (**A**) Splenic long-axis measurements on the coronal view of abdominal CT, the splenic long-axis larger than 15 cm. (**B**) Axial CT image showing massive splenomegaly. (**C**) Sagittal CT image showing massive splenomegaly.

Splenomegaly is a common clinical presentation in children with hematological disorders and immune diseases (1, 2), some common ones include thalassemia, hereditary spherocytosis, sickle cell anemia, and immune thrombocytopenic purpura, China is a high-incidence area for thalassemia, especially in Guangxi of Southwest China. Massive splenomegaly (Figure 1) carries risks of splenic rupture, and hypersplenism, and promotes disease progression. Hence, splenectomy plays an important role in controlling disease progression (3, 4).

Minimally invasive surgery is the direction of surgery, and the aim is to reduce the physical and psychologically traumatic stress of surgical patients, particularly pediatric patients. Laparoscopic surgery is more minimally invasive and aesthetically pleasing than traditional surgery. laparoscopic splenectomy (LS) has been a widely accepted technique for the removal of the spleen (5, 6). Most studies in LS have primarily been focused on adult patients, but only a few studies focus on splenomegaly children the operative field is poorly exposed because of the large spleen and small operating space in the abdominal cavity. In addition, uncontrollable massive bleeding easily occurs because of clinicopathological characteristics, such as tortuous and dilated vessels around the spleen, thin and brittle vessel walls, and high pressure in the lumen, thus making LS difficult to perform. Many scholars (7–10) have argued that single-incision laparoscopic surgery has the advantages of better cosmetic results and less trauma than multi-incision laparoscopic surgery. The recent development of the single-incision laparoscopic technique in the surgical field has achieved the prevention of visible postoperative scars. Thus, this technique is preferred by many patients. However, single-incision laparoscopy has not been widely used, and several complex surgeries have been abandoned because of technical challenges. Some studies (11, 12) have recently evaluated the effects of single-incision Laparoscopic Splenectomy (SILS) for the treatment of splenomegaly. However, SILS is rarely reported in children, due to limited operating space and the angle of surgery.

This was a prospective observational study, the safety, feasibility, advantages, and limitations of SILS in children were discussed by analyzing the data of 120 cases of pediatric patients who underwent SILS.

## Materials and methods

### Patients and clinical data collection

This study was approved by the Ethics Committee of the First Affiliated Hospital of Guangxi Medical University. This protocol complies with the declaration of Helsinki. We informed the patients of the operation process and related complications, and they signed a surgical consent before the surgery. All parents or guardians were informed about the operation process and possible complications in detail and written surgical consent was obtained.

Patients were recruited from the inpatient departments of pediatric surgery of the first affiliated hospital of Guangxi medical university. The inclusion criteria are as follows: (1) The patient must have a confirmed diagnosis of transfusion-dependent anemia. (2) The age of enrolled patients was greater than or equal to 5 years. (3) The annual blood transfusion volume is greater than 200 ml/kg. (4) The physical examination revealed massive splenomegaly, and the splenic long-axis size greater than 15 cm. Exclusion Criteria were a severe or unstable cardiopulmonary disease, and participation in another intervention trial.

A total of 120 pediatric patients underwent SILS for massive splenomegaly by the same surgeon at the department of pediatric surgery between October 2018 to September 2022. Computed tomography, abdominal ultrasound, and hematologic testing were performed for patients to evaluate spleen size and primary disease. All patients were confirmed to meet the inclusion criteria. The perioperative data were collected for descriptive statistics, including age, gender, height, weight, body mass index (BMI), splenic long-axis size, primary disease, operation time, intraoperative blood loss, postoperative pain score, anal exhaust time, postoperative hospital stay, and complications. We measured splenic axis size in computed tomography (CT) (see Figure 1 for details).

### Steps of surgical procedure

We developed a detailed surgical procedure and evaluation plan before the start of the study. All operations were performed by the same surgeon in our hospital. SILS was converted to multi-incision laparoscopic or open surgery when uncontrollable bleeding and severe operational difficulties occur during the operation.

**Figure 2:**
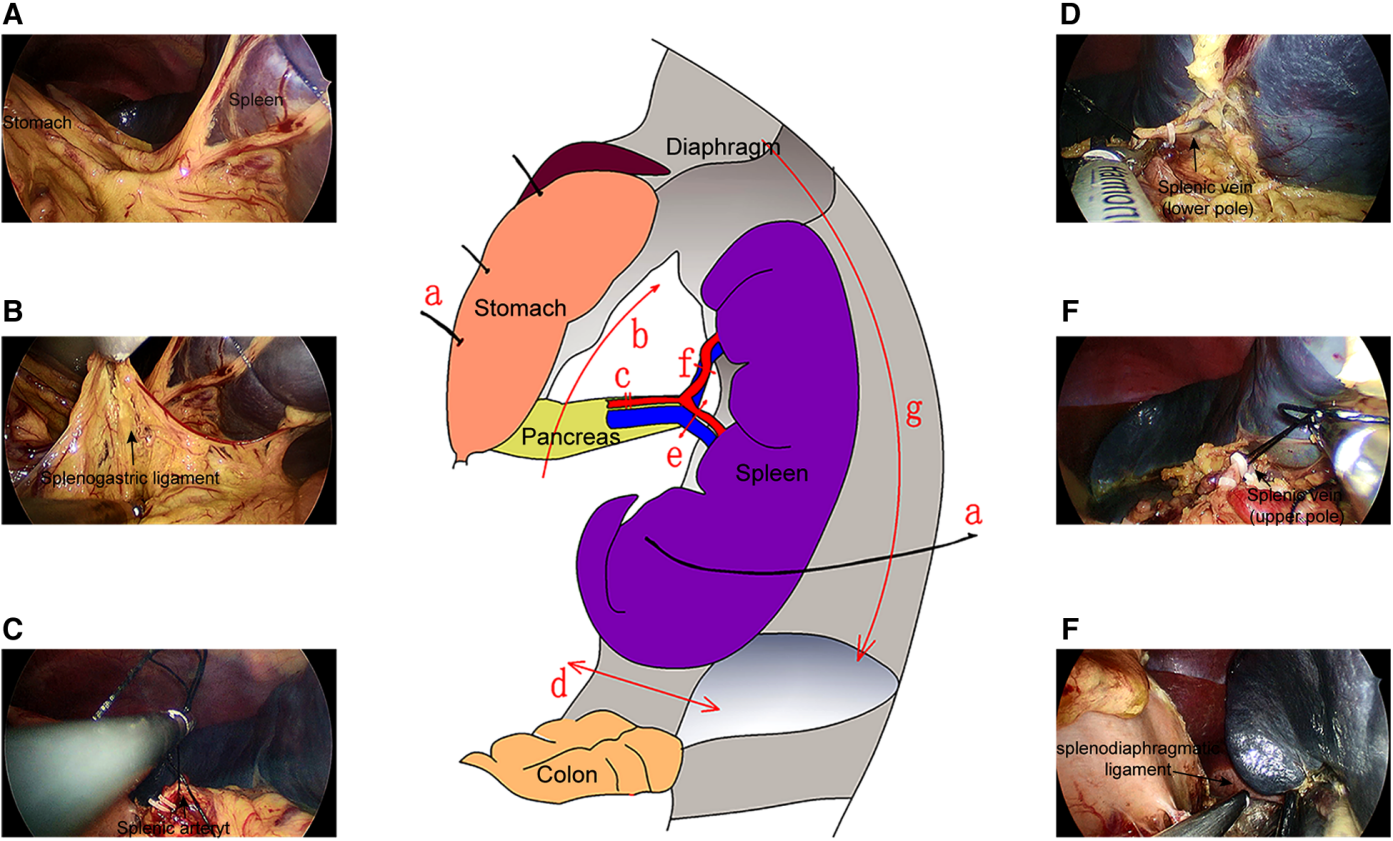
Operation flow of the SILS. (**A**) The traction sutures were used to pull the spleen and stomach, such that the splenogastric ligaments were in tension. (**B**) The splenogastric ligament was cut to expose the pancreatic tail and splenic hilum. (**C**) The splenic artery was exposed, divided, and ligated. The lower pole (**D**) and upper pole vein (**E**) of the spleen was exposed, ligated, and cut off. (**F**) The splenodiaphragmatic ligament and splenorenal ligament were severed.

**Figure 3:**
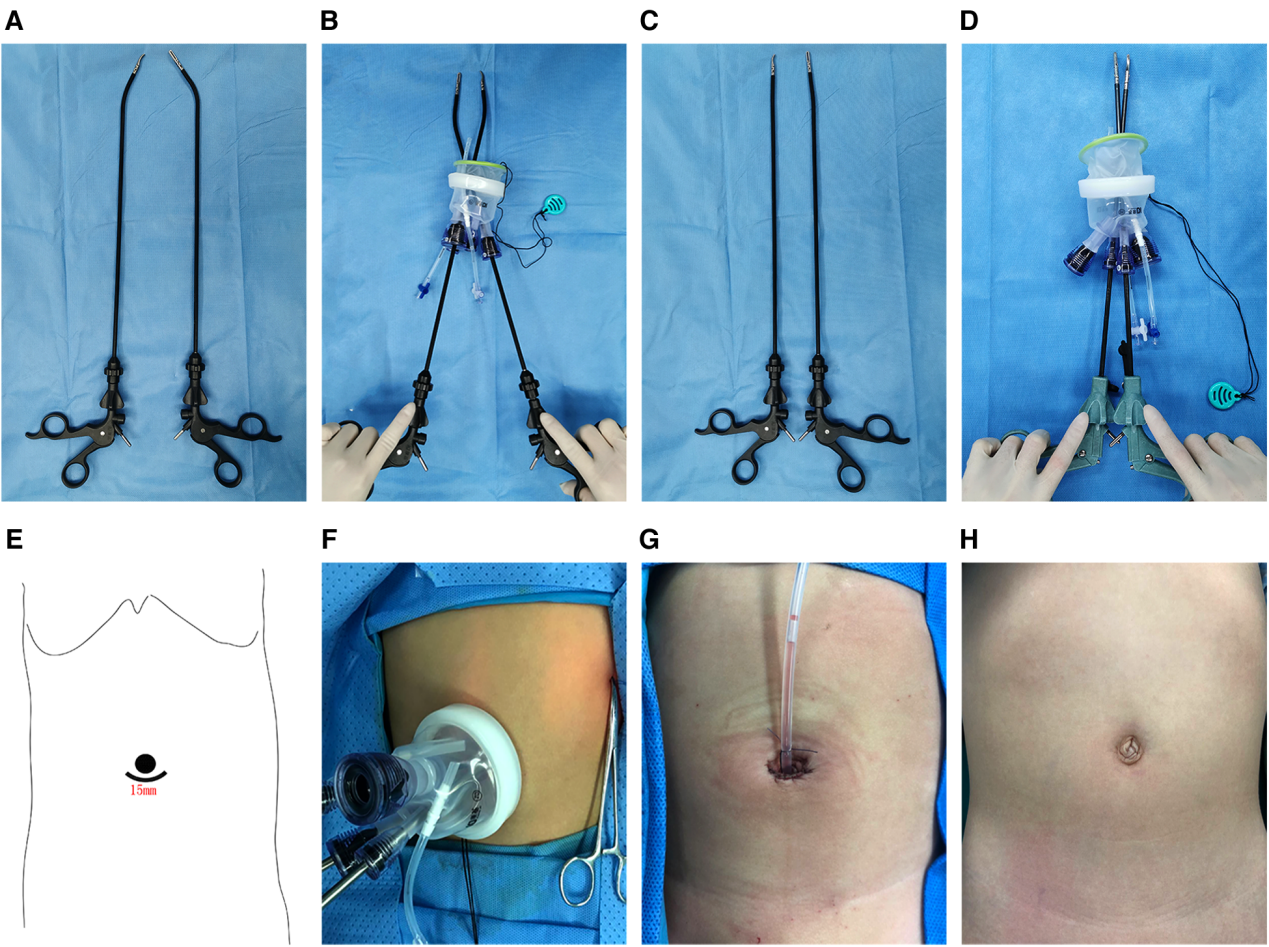
Surgical instrument system and incision design. (**A**) The parallel distribution of curved surgical instruments. (**B**) The crossover distribution of curved surgical instruments. (**C**) The parallel distribution of straight surgical instruments. (**D**) The crossover distribution of straight surgical instruments. (**E**) The surgical incision of SILS. (**F**) The SILS surgery port device. (**G**) Immediate postoperative image of the incision. (**H**) The appearance of the incision on 1 month after surgery.

The surgical procedure of SILS is detailed in Figure 2. Under general anesthesia, patients were placed in the supine position with the torso tilted 30° to the right. First, a 1.5 cm long (approximate) arc-shaped incision was made below the navel, The Single-port laparoscopic device will be implanted (Kanji, China), and CO_2_ pneumoperitoneum was established at 8–12 mmHg. A 5 mm rigid laparoscopy with a 30-degree camera lens was used. The stomach was pulled to the upper right side after two or three stitches of suspension suture were made on the stomach wall. The left lateral abdominal wall was sutured around the lower pole of the spleen, and the spleen was pulled to expose the splenic hilum. Then, an ultrasound scalpel was used to cut off the spleen-gastric ligament and the short gastric vessels. The splenic artery was dissociated and ligated. The splenic lower pole vessels were clipped and divided after the division of the splenocolic ligament. Subsequently, the splenic upper pole vessels were isolated and processed. The splenodiaphragmatic and splenorenal ligaments were detached using an ultrasound scalpel from top to bottom to dissociate the spleen completely. Thereafter, the spleen was crushed in a specimen bag and removed from the abdominal cavity, and a drainage tube was placed in the abdomen or pelvic cavity. Finally, the umbilical cord incision was closed (Figure 2). In our study, two types of Surgical instrument systems were used to complete the surgical procedure. The instrument system and incision design are in Figure 3.

### Postoperative management

After the operation, all patients were returned to the general ward. Nasogastric tubes were removed at the end of the operation. Four hours after resuscitation, the patient started on a clear liquid diet. After the first anal exhaust, the patients were suggested to gradually transition into a soft diet. On the first day after the surgery, the patients started to get out of bed with assistance. Routine postoperative pharmacological management included oral antiplatelet drugs which included aspirin and dipyridamole, started on the first postoperative day, and a long-acting antibiotic was used to prevent infection. The abdominal drainage tubes were removed 2–4 days after surgery. Hematology chemistry, physical examinations, and vital sign assessments were performed throughout treatment. All patients were followed up 1 month after surgery, the routine follow-up examinations included physical examination, hematology chemistry, and color doppler examinations. The last follow-up date was October 2022.

### Statistical methods

Statistical analysis was performed using Python 3.6.6. Enumeration data were expressed as median ± standard deviation (SD) or minimum and maximum (min; max). Mann–Whitney *U* test, *t*-test, and *χ*^2^-test of the categorization variable were used to evaluate the differences between the two groups. *P* < 0.05 was considered statistically significant.

## Results

### Patient demographics and clinical characteristics

The demographics and clinical characteristics of patients who underwent SILS (*n* = 120) are illustrated in Table 1. Their mean (±SD) age was 8.24 (±2.22) years, and their mean (±SD) height, weight, and BMI were 129 (±15.60) cm, 27.9 (±9.92) kg, and 16.2 (±2.55) kg/m^2^, respectively. Females were in the majority (59.17%, 49 males, 71 females). The most predominant primary disease involving massive splenomegaly was α-thalassemia (*n* = 95,79.17%) and followed by β-thalassemia (*n* = 21, 17.50%) and hereditary spherocytosis (*n* = 4, 3.33%). The mean (±SD) Splenic long-axis size on preoperative CT was 20.7 (±2.82) cm. There were no significant differences in preoperative clinical data between the SILS-S and SILS-C groups.

**Table 1:** The baseline and perioperative data of 120 patients undergoing SILS.

	Total (*n* = 120)	Success group^a^ (*n* = 103)	Failure group^b^ (*n* = 17)	*P*-value
Gender (%)				0.110
Female	71 (59.17%)	57 (55.34%)	14 (82.35%)	
Male	49 (40.83%)	46 (44.66%)	3 (17.65%)	
Age (SD), years	8.24 (2.22)	8.03 (2.17)	9.53 (2.12)	0.034*
Height (SD), cm	129 (15.60)	128 (14.40)	141 (17.40)	0.004*
Weight (SD), kg	27.9 (9.92)	26.8 (9.06)	34.7 (12.30)	0.010*
BMI (SD), kg/m^2^	16.2 (2.55)	16.1 (2.53)	16.9 (2.67)	0.456
Splenic long-axis (SD), cm	20.7 (2.82)	20.7 (2.86)	20.5 (2.67)	0.969
Primary disease (%)				0.896
α-thalassaemia	95 (79.17%)	82 (79.61%)	13 (76.47%)	
β-thalassaemia	21 (17.50%)	17 (16.50%)	4 (23.53%)	
Hereditary spherocytosis	4 (3.33%)	4 (3.89%)	0 (0%)	
Surgical protocol (%)				0.735
SILS-C^c^	60 (50.00%)	53 (51.46%)	7.00 (41.18%)	
SILS-S^d^	60 (50.00%)	50 (48.54%)	10.0 (58.82%)	
Operating time (SD), min	189 (48.90)	188 (48.70)	190 (52.05)	0.988
Blood loss (Min, Max), ml	20 (5, 425)	20 (5, 290)	320 (125, 425)	<0.001*
Pain score at Day 1 (SD)^e^	2.93 (0.95)	2.89 (0.91)	3.12 (1.17)	0.663
Pain score at Day 3 (SD)^e^	0.975 (0.88)	0.961 (0.89)	1.06 (0.89)	0.915
Anal exhaust time (SD), h	24.7 (8.79)	23.9 (7.73)	29.4 (12.90)	0.061
Postoperative hospital stay (SD), day	4.78 (1.03)	4.72 (1.03)	5.12 (0.99)	0.336
Complications (%)				
No	110 (91.67%)	95 (92.23%)	15 (88.24%)	0.858
Yes	10 (8.33%)	8 (7.77%)	2 (11.76%)	

^a^ Surgery was completed as planned.

^b^ Surgery was not completed as planned and intraoperative conversion to other surgical programs.

^c^ The SILS with curved surgical instrumentation.

^d^ The SILS with straight surgical instrumentation.

^e^ FLACC: The primary outcome measure will be the pain rating using the Faces, Legs, Activity, Cry and Consolability Scale during the procedure.

* *P* < 0.05.

### Risk factor for surgical failure of SILS

The patients were forced to change in surgical procedure, and these samples were defined as the failure group, and the success group was defined as the completion of the SILS operation. Among the 17 patients in the failure group, uncontrollable bleeding in 14 (82.35%) cases and severe operational difficulties in 3 (17.65%) cases. The baseline clinical data of patients were compared between the success and failure groups (Table 1). The results of the univariate analysis demonstrated that year, height and weight were the potential risk factor in surgery failure. Patients had a significantly greater age in the failure group than in the success group (9.53 ± 2.12 vs. 8.03 ± 2.17, *P* = 0.034), this tendency was observed in height (141 ± 17.40 vs. 128 ± 14.40, *P* = 0.004) and weight (34.7 ± 12.30 vs. 26.8 ± 9.06, *P* = 0.010). However, there was no significant difference in gender, BMI, and splenic long-axis. However, there was no independent risk factor for surgical failure in multivariate logistic analysis (Table 2).

**Table 2:** Multifactor logistics regression analysis of risk factors for surgical failure of SILS.

	OR value	OR 2.5%	OR 97.5%	*Z* value	*P*-value
Age, years	0.886	0.523	1.460	−0.469	0.639
Weight, kg	0.982	0.880	1.092	−0.333	0.739
Height, cm	1.087	0.978	1.216	1.518	0.129

### Operation data and postoperative results

All of the total enrolled patients had an uneventful postoperative course. The mean (±SD) operating time, pain score on day 1, pain score on day 3, time of first anal exhaust, and postoperative hospital stay were 189 (±48.90) minutes, 2.93 (±0.95), 0.975 (±0.88), 24.7 (±8.79) hours and 4.78 (±1.03) days, respectively. The median (min, max) blood loss was 15 (5, 365) ml. The operation data and postoperative results are reported in Table 1. The failure group included 4 patients with the open procedure and 13 patients with a multi-incision laparoscopic procedure. The mean (±SD) operation time of the success group was 188 (±48.70) min, and this result was consistent with the failure group. The median (min, max) intra-operative blood loss was 20 (5, 290), which was fewer than that for the failure group (*P* < 0.001). The two groups had no significant differences in pain scores, anal exhaust time, postoperative hospital stay, and postoperative complications (Table 1).

The 103 patients who underwent SILS were grouped as follows: 53 patients in the SILS-S group, and 50 patients in the SILS-C group. The two groups did not significantly differ in any of the clinical parameters examined. However, the mean (±SD) operation time was significantly shorter in the SILS-C group compared with the SILS-S group (172 ± 44.21 vs. 205 ± 47.90 min, *P* = 0.003), and there were no significant differences in other perioperative data (Table 3).

**Table 3:** The baseline and perioperative data of SILS-C versus SILS-S.

	SILS-C^a^ (*n* = 53)	SILS-S^b^ (*n* = 50)	*P*-value
Gender (%)			0.870
Female	28 (52.83%)	29 (58.00%)	
Male	25 (47.17%)	21 (42.00%)	
Age (SD), years	8.11 (2.26)	7.94 (2.08)	0.921
Height (SD), cm	129 (14.01)	126 (14.80)	0.501
Weight (SD), kg	28.3 (9.46)	25.2 (8.42)	0.229
BMI (SD), kg/m^2^	16.5 (2.66)	15.6 (2.31)	0.151
Splenic long-axis (SD), cm	20.2 (2.91)	21.3 (2.72)	0.158
Primary disease (%)			0.842
α-thalassaemia	44 (83.02%)	38 (76.00%)	
β-thalassaemia	8 (15.09%)	9 (18.00%)	
Hereditary spherocytosis	1 (1.89%)	3 (6.00%)	
Operating time (SD), min	172 (44.21)	205 (47.90)	0.003*
Blood loss (Min, Max), g	15 (5, 365)	20 (5, 290)	0.121
Pain score at Day 1 (SD)^c^	3.08 (0.90)	2.70 (0.89)	0.109
Pain score at Day 3 (SD)^c^	1.11 (0.91)	0.800 (0.83)	0.198
Anal exhaust time (SD), h	24.70 (6.41)	23.10 (8.91)	0.601
Postoperative hospital stay (SD), day	4.83 (1.07)	4.60 (0.99)	0.529
Complications (%)			0.713
No	50 (94.34%)	45 (90.00%)	
Yes	3 (5.67%)	5 (10.00%)	

^a^ The SILS with curved surgical instrumentation.

^b^ The SILS with straight surgical instrumentation.

^c^ FLACC: The primary outcome measure will be the pain rating using the Faces, Legs, Activity, Cry and Consolability Scale during the procedure.

* *P* < 0.05.

### Postoperative complications

The postoperative complications are summarized in Table 3. Complications after SILS surgery were experienced by 8.33% (10/120) of patients, and there were no deaths during the study. Overall postoperative complications included intra-abdominal bleeding (*n* = 3, 0.25%), incision infection (*n* = 3, 0.25%), intra-abdominal infection (*n* = 1, 0.83%), overwhelming post-splenectomy infection (*n* = 1, 0.83%), intestinal obstruction (*n* = 1, 0.83%), and portal vein thrombosis (*n* = 1, 0.83%). Postoperative intra-abdominal bleeding was defined as the outflow of red or dark red blood-tinged fluid from the abdominal drainage tube. In the SILS-S group, 1 patient underwent reoperation due to intra-abdominal infection 12 days after the SILS. The remaining complications were resolved with conservative treatment (Table 4).

**Table 4:** Postoperative complications of SILS.

Grades^a^	Complications	*N* (%)
II	Intra-abdominal bleeding	3 (0.25%)
I	Incision infection	3 (0.25%)
IIIb	Intra-abdominal infection	1 (0.83%)
IV	Overwhelming post-splenectomy infection	1 (0.83%)
II	Intestinal obstruction	1 (0.83%)
II	Portal vein thrombosis	1 (0.83%)

^a^ Clavien-Dindo classification.

## Discussion

In this pilot study, a SILS procedure was used as a new surgical scheme for massive Splenomegaly in children, who has been diagnosed with thalassemia or hereditary spherocytosis. Since 2018, our team has conducted SILS in 120 pediatric patients with massive splenomegaly in our center. Before this operation, we accumulated considerable experience in children's single-incision laparoscopic surgery. Here, we describe the findings of our study that surgical indicators, post-operative recovery, complications, and surgery completion rate. Overall, we found a surgery completion rate of 85.83% and the complication rate of 7.77%, and these results demonstrate that SILS also has technical feasibility and safety in children's spleen surgery. Moreover, the cosmetic advantage of SILS is unquestionable, SILS can minimize surgical trauma in children by preventing evident surgical scars. It can also considerably reduce physical and psychological trauma in children. A large number of studies (13–16) have confirmed that interference between instruments is one of the main difficulties of single-incision laparoscopic operation. Our study found the surgical failure rate of SILS may be associated with factors including the age, height, and weight of the patients. In our opinion, older age, greater height, and a larger body mass would indicate a deeper surgical site and that would increase the collision of instruments and surgical difficulty. But we were not able to find the same result by using multivariate linear regression analysis, which might be related to the small sample size.

In the clinical study, operation time and blood loss were important factors for assessing surgical difficulty and trauma. We found that the surgical difficulty of single-channel laparoscopic surgery is higher than that of traditional laparoscopic surgery. In the present study, the average operative time was 188 min, and the average volume of blood loss was 20 ml. This result is considerably higher than the operative time of LS (116.36–132.17 min) reported by Cetin et al. (17), but the blood loss was not significantly more than previously reported surgical results of LS (18–20). Single-incision laparoscopic procedures are performed by introducing all surgical instruments through a single incision in the navel. However, the major disadvantage of single-incision laparoscopic surgery is the mutual interference of surgical instruments, small operating space, and difficulty in surgical exposure. Safety and feasibility are key indicators of the applicability of technology, consequently, several technical issues have to be solved to be able to apply this technique in a clinical setting. During the surgical procedure, conduct the crossing of the left and right surgical instruments to overcome the collision between the endoscope and surgical instruments. also, curved laparoscopic instruments and high-definition endoscopes have been first introduced to increase the operative visual field. Under the same intraperitoneal operating distance, the distance between the handles of curved surgical instruments is significantly greater than that of straight surgical instruments, such a phenomenon is schematically depicted in Figures 3B,D. The study (21, 22) demonstrated that the collision of surgical instruments collision was reduced by crossing the left and right surgical instruments to ensure a triangular operation layout, and it can greatly reduce the difficulty of a single-incision laparoscopic approach and assured a relatively favorable field. In our second study, we compared the surgical parameters of two surgical instruments, and confirmed that the curved laparoscopic instruments could lead to a shorter operative time, but not decrease the intraoperative blood loss, recovery, and post-operative complications. The curved laparoscopic instrument reduces the interference of instruments, and this also increases operating space.

Intraoperative bleeding is the most common complication affecting the efficacy and prognosis of surgery and is a crucial factor. it is difficult to control bleeding in a timely fashion once the intraoperative bleeding occurs during laparoscopic surgery (19, 23, 24). In the present study, 14 of 120 patients experienced uncontrollable bleeding during operation, eleven cases experienced bleeding from the splenic vein, and another three patients experienced short gastric vessels. It was treated by conversion to open surgery in four and by conversion to multi-incision laparoscopic surgery in another ten. The surgical difficulty in massive splenomegaly is the management of splenic vessels, particularly the ligation and dissection of splenic veins. The volume of the enlarged spleen caused increased vascular blood flow and vessel diameter, Indeed, the increased blood flow of the splenic vein increases the stress on the vascular wall, rendering it more prone to rupture. Rupture and massive bleeding can easily occur during the separation process and affect the entire operation. Intraoperative bleeding is one of the major surgical risks in surgery failure of SILS, the operator's knowledge of the laparoscopic surgery should be proficient, and the intraoperative movements should be gentle to avoid damage to the splenic vessels. Some studies (25, 26) have reported that the use of Endo-GIA universal endoscopic vascular linear staplers resulted in significant reductions in intraoperative blood loss and operative time of laparoscopic splenectomy, however, this technology is limited by the angle and space of operation in this study.

The incidence rate of splenectomy complications was 1.19%–16.0% and included incision infection, intraperitoneal hemorrhage, visceral injury, intraperitoneal infection, intestinal obstruction, Pancreatic fistula, and systemic infection (27–32). In the present study, the overall postoperative complication rate was 8.33%. This result is consistent with the current literature, showing low complication rates for SILS (33–35). The main postoperative complications reported in our group were intra-abdominal bleeding and incision infection. The incision complications can be attributed to the incorrect selection of the incision, the incision being too close to the center of the navel, and the arc incision exceeding the half circle of the navel, all of which can result in poor blood circulation in the navel after the operation. Therefore, we suggest that the operative channel should be relaxed to ensure blood circulation in the navel if the operating time is extremely long. The spleen is a major immune organ in the body, the immunologic function may have been decreased and increase the risk of postoperative infection after splenectomy, it should be noted that overwhelming post-splenectomy infection (OPSI) after splenectomy, is highly lethal, with an estimated mortality rate of 30%–50% (36, 37). In the current study, one patient underwent reoperation for severe intraabdominal infections. We speculated that the reason is the low immunity and intra-abdominal fluid. Thus, to prevent infections, intravenous antibiotic prophylaxis may be necessary, especially in childhood. Portal vein thrombosis (PVT) is a serious complication of splenectomy, with a prevalence rate ranging from 10% to 55.7%, and the Splenic volume and splenic vein diameter are independent pre-operative risk factors of PTV after splenectomy (38–41). It is a possible cause of platelet and the decrease of decreasing portal blood flow was responsible for the development of thrombus. The incidence of PVT in the current study was 0.73%, which is lower than those reported in the literature. We consider that the above results are related to the routine use of anticoagulants.

In conclusion, SILS is safe and feasible for children with massive splenomegaly, and the best cosmetic outcomes will reduce the physical and mental trauma of patients. However, it is our opinion that the difficulty of the surgery can be reduced by using curved laparoscopic instruments.

## Data Availability

The original contributions presented in the study are included in the article/Supplementary Material, further inquiries can be directed to the corresponding authors.
